# Acute post-stroke blood pressure relative to premorbid levels in intracerebral haemorrhage versus major ischaemic stroke: a population-based study

**DOI:** 10.1016/S1474-4422(14)70031-6

**Published:** 2014-04

**Authors:** Urs Fischer, Marie Therese Cooney, Linda M Bull, Louise E Silver, John Chalmers, Craig S Anderson, Ziyah Mehta, Peter M Rothwell

**Affiliations:** a Stroke Prevention Research Unit, Nuffield Department of Clinical Neurosciences, University of Oxford, Oxford, UK; b The George Institute for Global Health, University of Sydney, Australia

## Abstract

**Background:**

It is often assumed that blood pressure increases acutely after major stroke, resulting in so-called post-stroke hypertension. In view of evidence that the risks and benefits of blood pressure-lowering treatment in acute stroke might differ between patients with major ischaemic stroke and those with primary intracerebral haemorrhage, we compared acute-phase and premorbid blood pressure levels in these two disorders.

**Methods:**

In a population-based study in Oxfordshire, UK, we recruited all patients presenting with stroke between April 1, 2002, and March 31, 2012. We compared all acute-phase post-event blood pressure readings with premorbid readings from 10-year primary care records in all patients with acute major ischaemic stroke (National Institutes of Health Stroke Scale >3) versus those with acute intracerebral haemorrhage.

**Findings:**

Of 653 consecutive eligible patients, premorbid and acute-phase blood pressure readings were available for 636 (97%) individuals. Premorbid blood pressure (total readings 13 244) had been measured on a median of 17 separate occasions per patient (IQR 8–31). In patients with ischaemic stroke, the first acute-phase systolic blood pressure was much lower than after intracerebral haemorrhage (158·5 mm Hg [SD 30·1] *vs* 189·8 mm Hg [38·5], p<0·0001; for patients not on antihypertensive treatment 159·2 mm Hg [27·8] *vs* 193·4 mm Hg [37·4], p<0·0001), was little higher than premorbid levels (increase of 10·6 mm Hg *vs* 10-year mean premorbid level), and decreased only slightly during the first 24 h (mean decrease from <90 min to 24 h 13·6 mm Hg). By contrast with findings in ischaemic stroke, the mean first systolic blood pressure after intracerebral haemorrhage was substantially higher than premorbid levels (mean increase of 40·7 mm Hg, p<0·0001) and fell substantially in the first 24 h (mean decrease of 41·1 mm Hg; p=0·0007 for difference from decrease in ischaemic stroke). Mean systolic blood pressure also increased steeply in the days and weeks before intracerebral haemorrhage (regression p<0·0001) but not before ischaemic stroke. Consequently, the first acute-phase blood pressure reading after primary intracerebral haemorrhage was more likely than after ischaemic stroke to be the highest ever recorded (OR 3·4, 95% CI 2·3–5·2, p<0·0001). In patients with intracerebral haemorrhage seen within 90 min, the highest systolic blood pressure within 3 h of onset was 50 mm Hg higher, on average, than the maximum premorbid level whereas that after ischaemic stroke was 5·2 mm Hg lower (p<0·0001).

**Interpretation:**

Our findings suggest that systolic blood pressure is substantially raised compared with usual premorbid levels after intracerebral haemorrhage, whereas acute-phase systolic blood pressure after major ischaemic stroke is much closer to the accustomed long-term premorbid level, providing a potential explanation for why the risks and benefits of lowering blood pressure acutely after stroke might be expected to differ.

**Funding:**

Wellcome Trust, Wolfson Foundation, UK Medical Research Council, Stroke Association, British Heart Foundation, National Institute for Health Research.

## Introduction

Blood pressure is increased in about 75% of patients with acute stroke (post-stroke hypertension) and usually decreases spontaneously over the subsequent few days.^1^, ^2^, ^3^, ^4^, ^5^, ^6^, ^7^, ^8^, ^9^, ^10^ Blood pressure in patients with acute stroke is higher than in those with other acute illnesses.^2^, ^7^, ^11^ However, a history of premorbid hypertension is also more common in patients with stroke than in otherwise healthy people,^2^, ^7^ and is associated with high post-stroke blood pressure^1^, ^2^, ^4^, ^5^, ^6^, ^8^, ^9^, ^12^ and more variable blood pressure.^13^ Blood pressure is assumed to increase as a consequence of stroke, possibly due to disturbed autoregulation,^14^ damage or compression of brain regions that regulate the autonomic nervous system,^15^ neuroendocrine factors,^16^, ^17^, ^18^ or as a consequence of headache,^19^ urine retention,^14^ infection,^19^ or the psychological stress of admission to hospital.^6^, ^12^ However, to date, acute post-stroke blood pressure levels have not been systematically compared with actual premorbid blood pressure levels.

The reduction of blood pressure after acute stroke has been shown to be either of no benefit or of slight harm in trials in predominantly ischaemic stroke,^20^, ^21^, ^22^, ^23^, ^24^, ^25^ but to be of some benefit in trials in primary intracerebral haemorrhage.^26^, ^27^, ^28^ However, the absence of a clear physiological rationale to explain this apparent difference in effect makes interpretation of the trial results difficult. We and others have hypothesised that any benefits of treatment to lower blood pressure in acute stroke might be greater in patients in whom a high post-event level is unaccustomed,^29^, ^30^ and we have suggested that in some patients post-stroke hypertension might be due to a recent premorbid increase in blood pressure.^31^ To better understand the nature of acute post-stroke hypertension in intracerebral haemorrhage versus ischaemic stroke, we aimed to determine the relation between premorbid and acute post-event blood pressure in these two subtypes of stroke in the Oxford Vascular Study (OXVASC) study.

## Methods

### Study design and participants

Details of the study design have been previously described.^32^, ^33^ Briefly, all patients presenting with stroke or transient ischaemic attack in Oxfordshire from April 1, 2002, to March 31, 2012, were recruited and followed up, irrespective of mode of presentation or referral (ie, emergency department, referred for direct admission to hospital, referred to clinic, or managed at home by the primary-care physician). Recruitment was achieved by several overlapping methods of daily hot and cold pursuit (appendix p 1), including prospective daily searches for acute events and retrospective searches of hospital and primary-care administrative and diagnostic coding data.^32^, ^33^, ^34^ As reported previously, more than 90% of patients were identified and assessed prospectively by hot pursuit,^34^ and overall case ascertainment is near-complete.^32^, ^33^

All patients gave written informed consent; a relative of those who were unable to provide consent gave informed assent. OXVASC received ethics approval from participating ethics review committees.

### Procedures

Patients were assessed by a study clinician as soon as possible after the event and informed consent (or assent from a relative) was obtained. Clinical assessments and initial diagnoses were made by clinical fellows and reviewed by a stroke neurologist. We used standard definitions of stroke.^32^ We recorded details of the presenting event by interviewing patients' relatives and family practitioner, and by checking primary care and hospital records. We also recorded medication use before the event, previous vascular events, and vascular risk factors. We obtained detailed data for times and methods of first contact with medical attention after the event using a structured questionnaire. Time of symptom onset was the moment when symptoms were first noticed by the patient. When patients were found unconscious or aphasic, we took the time when they were last seen without symptoms as the start of the event. For symptoms present on waking, we took the time of waking as the time of symptom onset, but we also did sensitivity analyses excluding such cases.

We recorded the first post-stroke blood pressure measurement in the emergency department for all patients. If a patient was first seen by their family doctor, we obtained post-stroke blood pressure values and exact time of recording from their medical records or referral documentation. If patients were first assessed by paramedics outside a care setting, we obtained the first blood pressure recorded by the paramedics by systematically searching all relevant records in the ambulance service headquarters or using the copy of the ambulance sheet in the patient's medical notes. In patients assessed hyperacutely (first blood pressure reading <90 min after symptom onset), we recorded all measurements during the first 24 h after the event.

We assessed the severity of neurological deficit with the National Institutes of Health Stroke Scale (NIHSS) score.^35^ Patients routinely underwent CT or MRI, carotid Doppler scanning, and electrocardiography, and had an echocardiogram when necessary. In view of the high rate (97%) of brain imaging or autopsy in OXVASC, and in keeping with our previous analyses,^32^, ^33^ we coded strokes of unknown type as ischaemic.^32^ We used the TOAST (Trial of Org 10172 in Acute Stroke Treatment) criteria to classify strokes into cause subtypes.^36^

In accordance with European guidelines at the time of the study,^37^, ^38^ blood pressure in the acute stage of the event was usually only lowered if systolic blood pressure exceeded 220 mm Hg or diastolic blood pressure exceeded 120 mm Hg. We documented any new blood pressure-lowering drug given within 24 h after the stroke. Any acute treatment was tailored to the individual, but usual policy was intravenous infusion of labetalol or nimodipine.

Study nurses reviewed life-long patient records held in primary care and extracted all premorbid blood pressure readings with dates in the 10 years before the event and the highest ever reading recorded in a standardised manner. We extracted data from both paper and computer records. Most readings had been taken in the doctor's surgery by the physician or the practice nurse, either for screening purposes, regular review, or an episode of minor illness. Measurements made during previous hospital admissions, often for major illness, were not recorded. We also excluded measurements made in primary care at the time of any previous transient ischaemic attack or stroke.

### Statistical analysis

We included all patients with primary intracerebral haemorrhage or major ischaemic stroke (NIHSS >3) who sought medical attention within 7 days after symptom onset. However, data for patients assessed hyperacutely (time from symptom onset to first blood pressure reading <90 min) and acutely (first reading <3 h after symptom onset) were analysed separately. If a patient had more than one event within the study period, we included the first event. We excluded patients if their strokes occurred when in a country other than the UK and data were not available.

We excluded patients with transient ischaemic attack or minor stroke because such patients often seek medical care after a substantial delay, because previous randomised trials of acute blood pressure-lowering have focused on major stroke,^20^, ^21^, ^22^, ^23^, ^24^, ^25^, ^26^, ^27^, ^28^ and to avoid a major disparity in the severity of ischaemic events versus intracerebral haemorrhage.

Using the data extracted from primary care records, we established the most recent premorbid blood pressure, the 10-year mean premorbid blood pressure, and the highest ever (maximum) premorbid blood pressure in patients with ischaemic stroke versus those in patients with primary intracerebral haemorrhage. We also established premorbid visit-to-visit variability of systolic blood pressure expressed as standard deviation (SD), coefficient of variation (CV), and variation independent of mean (VIM), as described previously.^39^ We identified any evidence of a systematic rise in blood pressure during the year before the stroke using regression analysis of the most recent premorbid blood pressure versus the log of the time from blood pressure measurement to stroke, stratified by stroke type. We repeated this analysis using the following blood pressure parameters: most recent recording minus second-most recent reading, most recent recording minus maximum reading, and most recent recording minus 10-year mean reading. We used the log of time from blood pressure reading to stroke because any premorbid increases in blood pressure before stroke were expected to cluster in the days and weeks immediately before the event.^31^

We compared the first post-event blood pressure with the most recent premorbid blood pressure, 10-year mean premorbid blood pressure, and the maximum premorbid blood pressure in patients with ischaemic stroke versus those in patients with intracerebral haemorrhage. Analyses were repeated by TOAST classification for ischaemic stroke and location (lobar or deep posterior) for intracerebral haemorrhage, by history of previous hypertension and by prior use of antihypertensives.

We compared pre-event and post-event readings using a paired *t* test. We used a *Z* test to test whether the percentage of post-event blood pressure readings greater than premorbid blood pressure readings was significantly different from 50%. We assessed differences in post-stroke blood pressure in subgroups with unpaired *t* tests or analysis of variance for continuous variables, and Fisher's two-tailed test for categorical variables. The population distributions of measures of blood pressure were represented graphically by kernel density estimates, which estimate the smoothed frequency distribution of measurements.

In patients who presented within 90 min of stroke onset, we analysed blood pressure trends over the subsequent 24 h. A regression line with patients as a random effect was fitted to estimate a random regression coefficient. We did this separately for patients with intracerebral haemorrhage and for those with major ischaemic stroke, excluding patients who received any additional blood pressure-lowering treatment.

We also compared the first post-event blood pressure with the most recent and the maximum pre-event readings in the Oxford Community Stroke Project (OCSP), a population-based study in the same population in Oxfordshire, UK, between 1981 and 1986. The methods of OCSP are described elsewhere.^40^ OCSP used similar methods of case ascertainment and premorbid blood pressure collection to OXVASC, but the first post-event blood pressure recorded was that measured when the patient was first seen by the study neurologist rather than at the first contact with medical or paramedical services. We used SPSS (version 20) for statistical analyses.

### Role of funding source

The sponsor of the study had no role in study design, data collection, data analysis, data interpretation, or writing of the report. The corresponding author had full access to all the data in the study and had final responsibility for the decision to submit for publication.

## Results

Of potentially eligible patients with stroke (n=734), 12 did not seek medical attention within 7 days, 12 were not referred to secondary care and had no acute blood pressure recorded, 16 died before any recording, 20 had their event in another country, and seven had it during cardiac arrest, inotropic support, or anaesthesia. A further 14 patients had intracerebral haemorrhage due to tumour, arteriovenous malformation, or another non-primary cause, leaving 653 patients with initial NIHSS >3, of whom 636 (97%) had premorbid readings. Differences in baseline demographic and clinical characteristics between patients with intracerebral haemorrhage and those with ischaemic stroke were as expected (table 1; appendix p 2).

**Table 1:** Baseline demographic and clinical characteristics

		**Major ischaemic stroke (N=523)**	**Intracerebral haemorrhage (N=113)**	**p value**
Mean age, years (SD)		78·7 (11·0)	75·9 (12·7)	0·02
Men		228 (44%)	60 (53%)	0·08
Diabetes		76 (15%)	11 (10%)	0·23
Smoking				0·95
	Never	269 (51%)	57 (50%)	..
	Ex-smoker	205 (39%)	46 (41%)	..
	Current smoker	49 (9%)	10 (9%)	..
Previous hypertension		352 (67%)	67 (59%)	0·13
Previous atrial fibrillation		169 (32%)	23 (20%)	0·01
Previous myocardial infarction		84 (16%)	6 (5%)	0·002
Previous angina		107 (20%)	12 (11%)	0·016
Previous transient ischaemic attack		75 (14%)	16 (14%)	1·00
Previous stroke		98 (19%)	11 (10%)	0·02
Previous peripheral vascular disease		58 (11%)	7 (6%)	0·17
Medication				
	Prior antihypertensives	352 (67%)	54 (48%)	0·0001
	Prior statin	144 (28%)	23 (20%)	0·13
	Prior antiplatelet therapy	258 (49%)	33 (29%)	0·0001
Number of premorbid BP readings				
	Mean (SD)	21·2 (16·7)	19·2 (15·0)	0·26
	Median (IQR)	17 (8–31)	16 (7–32)	0·32
Stroke cause (TOAST subtype)				
	Large artery disease	37 (7%)	..	..
	Cardioembolic	211 (40%)	..	..
	Small artery disease	48 (9%)	..	..
	Other determined cause	15 (3%)	..	..
	Undetermined cause	77 (15%)	..	..
	Unknown cause	120 (23%)	..	..
	More than one cause	15 (3%)	..	..
Location of intracerebral haemorrhage				
	Lobar	..	45 (40%)	..
	Deep or posterior	..	66 (58%)	..
	Both lobar and deep	..	1 (1%)	..
	Unknown	..	1 (1%)	..

Data are n (%) unless otherwise specified. BP=blood pressure. TOAST=Trial of Org 10172 in Acute Stroke Treatment.

Premorbid blood pressure (total readings 13 244) had been measured on a median of 17 separate occasions per patient (IQR 8–31). The number of premorbid readings was similar for intracerebral haemorrhage and ischaemic stroke (table 1), as was the distribution of time from most recent reading to stroke onset (appendix p 3). We noted a positive correlation between the number of premorbid readings and the mean premorbid systolic blood pressure (appendix p 4), with the association present in both intracerebral haemorrhage and ischaemic stroke.

The 10-year mean premorbid systolic blood pressure was much the same for patients with ischaemic stroke and those with intracerebral haemorrhage (table 2), but maximum premorbid systolic blood pressure was higher in patients with ischaemic stroke than in those with intracerebral haemorrhage (mean difference 6·5 mm Hg, 95% CI 0·5–12·4; p=0·03; table 2), as was premorbid visit-to-visit variability (SD 16·89 mm Hg *vs* 15·13 mm Hg, p=0·01; CV 11·29% *vs* 10·11%, p=0·006; VIM 11·42 *vs* 9·96, p=0·0004).

**Table 2:** Systolic blood pressure, measured before and immediately after event, by stroke subtypes

		**Patients assessed within 3 h**					**All patients**				
		n	Maximum premorbid (mm Hg)	10-year mean premorbid (mm Hg)	Most recent premorbid (mm Hg)	First post event (mm Hg)	n	Maximum premorbid (mm Hg)	10-year mean premorbid (mm Hg)	Most recent premorbid (mm Hg)	First post event (mm Hg)
Ischaemic stroke		294	179·7 (28·6)	147·9 (15·2)	140·6 (20·5)	158·5 (30·1)	523	180·9 (29·6)	148·6 (15·8)	140·8 (21·3)	158·1 (30·5)
Intracerebral haemorrhage		68	177·7 (26·1)	149·1 (17·9)	146·3 (22·5)	189·8 (38·5)	113	174·5 (27·2)	147·7 (17·7)	143·9 (22·1)	182·8 (37·6)
No previous hypertension											
	Ischaemic stroke	94	158·4 (21·8)	140·4 (14·0)	133·8 (17·9)	150·3 (28·1)	171	160·5 (23·1)	140·2 (14·1)	135·2 (18·1)	151·3 (29·8)
	Intracerebral haemorrhage	23	161·7 (23·4)	140·2 (14·9)	142·2 (18·8)	191·4 (35·8)	46	157·4 (24·4)	138·8 (16·1)	139·5 (16·9)	176·5 (36·2)
Previous hypertension											
	Ischaemic stroke	200	189·7 (25·9)	151·1 (14·7)	143·8 (20·9)	162·3 (30·4)	352	190·9 (27·1)	152·3 (15·1)	143·5 (22·1)	161·4 (30·4)
	Intracerebral haemorrhage	45	186·0 (23·7)	153·2 (17·8)	148·4 (24·1)	189·0 (40·1)	67	186·1 (22·5)	153·1 (16·5)	147·0 (24·6)	187·1 (38·2)
Location of intracerebral haemorrhage											
	Lobar	26	164·1 (21·9)	140·6 (16·0)	142·5 (20·1)	178·5 (32·0)	45	164·8 (24·7)	142·5 (16·5)	141·6 (19·0)	174·5 (34·2)
	Deep or posterior	40	186·7 (25·6)	154·8 (17·5)	149·4 (24·2)	193·8 (38·9)	66	181·0 (27·4)	151·3 (18·0)	145·8 (24·1)	186·2 (37·3)
TOAST subtype											
	Cardioembolic	132	180·4 (29·1)	148·3 (15·5)	140·5 (21·4)	159·5 (30·2)	211	183·1 (29·7)	148·7 (15·7)	139·1 (21·5)	159·0 (31·0)
	Large artery	26	186·3 (26·4)	148·7 (13·7)	141·0 (23·5)	154·6 (26·6)	37	186·4 (26·7)	150·5 (14·8)	145·8 (26·6)	160·9 (28·8)
	Small vessel	14	170·6 (26·5)	146·8 (16·6)	145·6 (29·2)	173·3 (33·8)	48	175·7 (28·5)	148·6 (17·7)	146·7 (23·8)	169·4 (27·3)
	Undetermined	40	175·0 (30·5)	145·8 (17·9)	139·4 (17·1)	152·5 (30·4)	77	179·2 (30·0)	148·1 (17·7)	141·0 (17·4)	157·7 (29·1)
	Unknown	64	183·7 (24·9)	149·7 (12·2)	141·4 (18·3)	160·3 (31·2)	120	180·4 (29·3)	148·9 (14·1)	139·9 (20·5)	153·1 (32·8)
	More than one cause	8	168·5 (30·2)	138·3 (16·4)	129·1 (14·2)	150·5 (25·4)	15	176·9 (28·5)	142·6 (14·8)	137·1 (21·0)	147·9 (21·6)
	Other known cause	10	167·7 (39·4)	147·3 (21·0)	141·7 (18·8)	152·4 (24·6)	15	170·4 (36·8)	147·7 (18·6)	142·1 (16·3)	154·3 (23·8)

Data are mean (SD). TOAST=Trial of Org 10172 in Acute Stroke Treatment.

**Figure 1:**
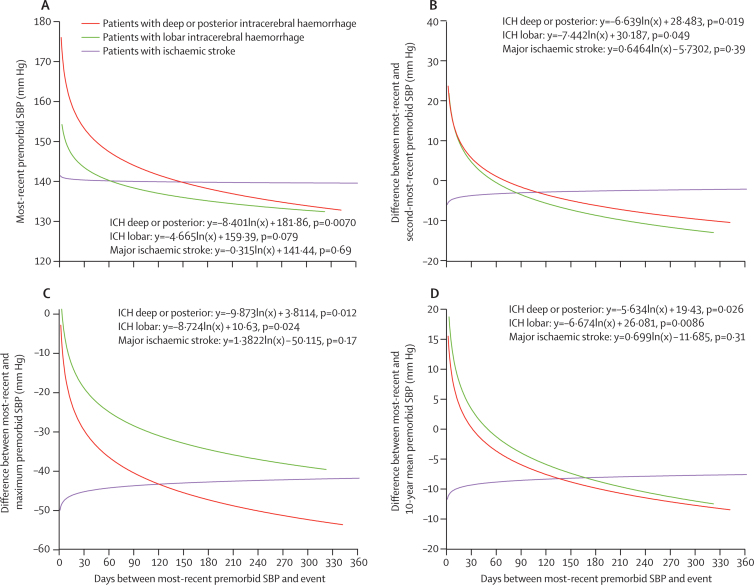
Temporal trends in premorbid systolic blood pressure measurements in relation to the time of stroke Most-recent systolic blood pressure (SBP; A), difference between most-recent and second-most-recent premorbid SBP (B), difference between most-recent and maximum premorbid SBP (C), and difference between most-recent and 10-year mean premorbid SBP (D). The lines and associated equations are derived from a log-linear regression. ICH=intracerebral haemorrhage.

Whereas the most recent premorbid systolic blood pressure in patients with ischaemic stroke was no greater in the period immediately before the stroke, systolic blood pressure was higher in the weeks and days before intracerebral haemorrhage (p<0·0001), especially in patients with deep or posterior bleeds (figure 1). We saw the same temporal pattern for the difference between the most recent and second-most recent systolic blood pressure (ie, the most recent premorbid rise on repeated measurements) and for the most recent premorbid systolic blood pressure versus the 10-year mean systolic blood pressure and the maximum premorbid systolic blood pressure (figure 1).

The median interval from stroke onset to first blood pressure measurement was 2·0 h (IQR 0·7–7·0) after intracerebral haemorrhage and 2·3 h (0·7–11·6) after ischaemic stroke. The first post-event blood pressure reading was done by a primary care physician for 51 (8%) patients, by paramedics or ambulance personnel in 337 (53%) patients, by emergency department personnel in 172 (27%) patients, and by others in 76 (12%) patients, with no difference between intracerebral haemorrhage and major ischaemic stroke (p=0·76). We detected no correlation between the first post-event systolic blood pressure and first NIHSS score in patients with intracerebral haemorrhage (*r*=0·02, p=0·85) or in those with ischaemic stroke (*r*=–0·02, p=0·67).

**Figure 2:**
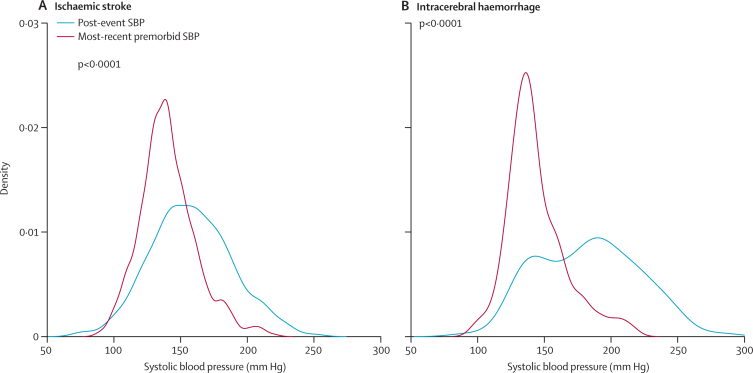
Distribution of most recent premorbid systolic blood pressure and first post-event systolic blood pressure (A) Ischaemic stroke. (B) Intracerebral haemorrhage.

For blood pressure measured within 3 h of stroke onset, mean first systolic blood pressure after intracerebral haemorrhage was higher than the most recent premorbid reading (average increase 43·5 mm Hg, p<0·0001; Table 2, Table 3, figure 2). The first systolic blood pressure after ischaemic stroke was lower than that after intracerebral haemorrhage (p<0·0001) and only a little higher than premorbid levels (a 17·9 mm Hg increase *vs* most-recent systolic blood pressure and a 10·6 mm Hg increase *vs* 10-year mean premorbid level; table 2, figure 2). Findings were similar in patients whose first measurement was taken more than 3 h after an event, on analysis excluding wake-up strokes, and on analysis restricted to patients with a first blood pressure reading within 6 h of stroke and at least two premorbid measurements within the previous 12 months (appendix pp 5–6). The differences in acute systolic blood pressure between intracerebral haemorrhage and ischaemic stroke were greatest in patients assessed within minutes of onset; mean post-event systolic blood pressure decreased with time from event after intracerebral haemorrhage, but not after ischaemic stroke (table 3).

**Table 3:** Relation between time to assessment and mean systolic blood pressure before and after event

	**0–15 min**	**15–30 min**	**30–90 min**	**90 min to 3 h**	**3–12 h**	**12–24 h**	**>24 h**
**Mean post-event SBP (mm Hg)**							
Intracerebral haemorrhage	204·4 (31·5); 8	192·0 (47·4); 18	188·9 (34·6); 25	181·9 (37·5); 17	173·8 (33·1); 24	172·0 (29·3); 11	168·7 (43·3); 10
Ischaemic stroke	155·0 (29·2); 42	156·0 (29·7); 54	159·6 (30·9); 119	160·4 (30·0); 79	156·5 (31·5); 103	157·9 (30·5); 50	158·8 (31·1); 76
**Mean increase in post-event SBP compared with most-recent premorbid SBP (mm Hg)**							
Intracerebral haemorrhage	46·0 (45·8); 8	47·5 (48·0); 18	44·1 (30·3); 25	37·1 (34·4); 17	36·9 (36·8); 24	26·5 (37·0); 11	25·9 (31·3); 10
Ischaemic stroke	11·8 (29·5); 42	20·6 (30·4); 54	18·6 (31·3); 119	18·2 (31·4); 79	19·3 (31·9); 103	13·1 (39·1); 50	15·3 (27·0); 76

Data are mean (SD); n. SBP=systolic blood pressure.

Although premorbid systolic blood pressure rose before intracerebral haemorrhage (figure 1), the additional increase from the most recent premorbid blood pressure reading to the first post-event reading was no less in patients with very recent premorbid readings than in those with readings taken a longer period before the event (p=0·63 for regression of increment *vs* time since reading). In other words, the high post-event systolic blood pressure in intracerebral haemorrhage was due to both a rise in systolic blood pressure premorbidly and a subsequent additional increase in systolic blood pressure from the last premorbid reading that was greater than that seen in ischaemic stroke, in which there was no premorbid increase and only a small post-event increase.

**Figure 3:**
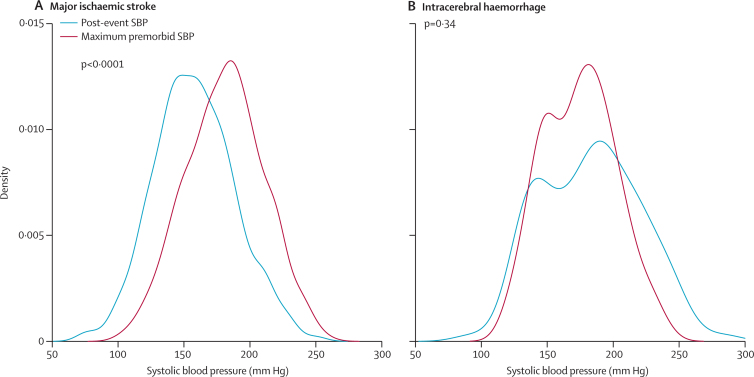
Distribution of maximum premorbid systolic blood pressure and first post-event systolic blood pressure in all patients (A) Ischaemic stroke. (B) Intracerebral haemorrhage.

Figure 2 shows the relative distributions of the most recent premorbid systolic blood pressure and the first post-event systolic blood pressure. The first post-event systolic blood pressure was higher than the 10-year average premorbid systolic blood pressure in only 304 (58%) of 523 patients with ischaemic stroke compared with 96 (85%) of 113 patients with intracerebral haemorrhage (p<0·0001). Figure 3 shows a comparison of the distribution of the first post-event systolic blood pressure with the maximum premorbid systolic blood pressure. 385 (74%) patients with ischaemic stroke had at least one higher office reading (reading in primary care) in the past. The first post-event systolic blood pressure exceeded the maximum pre-event level in only 63 (28%) of 229 patients with severe ischaemic stroke (NIHSS >10). By contrast, the first post-event systolic blood pressure reading was higher than the maximum-ever premorbid level in 62 (55%) of 113 patients with primary intracerebral haemorrhage. Results were similar for diastolic blood pressure (data not shown).

**Figure 4:**
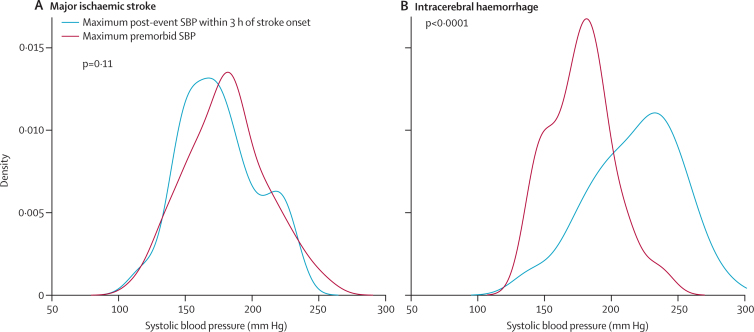
Distribution of maximum premorbid systolic blood pressure and maximum systolic blood pressure within 3 h after the event in patients presenting within 90 min of stroke onset (A) Ischaemic stroke. (B) Intracerebral haemorrhage.

Because a patient's first post-event systolic blood pressure might not be their highest acute-phase systolic blood pressure, in those patients who were assessed within 90 min of stroke we compared the highest post-event systolic blood pressure reading measured within 3 h of symptom onset with the maximum premorbid blood pressure (figure 4). Maximum pre-event and post-event systolic blood pressure were similar after ischaemic stroke (mean 180·6 mm Hg *vs* 175·4 mm Hg, mean difference 5·2, 95% CI −1·2 to 11·6, p=0·16), but the average post-event maximum was substantially greater after primary intracerebral haemorrhage (217·6 mm Hg *vs* 177·6 mm Hg, mean difference 50·0, 38·1 to 61·8, p<0·0001). We detected no difference between intracerebral haemorrhage and ischaemic stroke in the timing of measurements in the first 24 h after stroke (appendix p 7).

The extent to which the first post-event systolic blood pressure exceeded premorbid levels is shown in table 4. The first acute-phase blood pressure was more likely to be the highest ever recorded in patients with primary intracerebral haemorrhage than in those with ischaemic stroke (OR 3·4, 95% CI 2·3–5·2, p<0·0001) and to be more than 40 mm Hg higher than the most recent premorbid reading (3·7, 2·4–5·6, p<0·0001) or the 10-year average (4·4, 2·8–7·1, p<0·0001), for primary intracerebral haemorrhage versus ischaemic stroke.

**Table 4:** Increase in systolic blood pressure after stroke in comparison with three measures of premorbid blood pressure control in intracerebral haemorrhage versus ischaemic stroke

	**Most-recent premorbid SBP**			**10-year average premorbid SBP**			**Maximum premorbid SBP**		
	Odds ratio (95% CI)	Intracerebral haemorrhage (n)	Ischaemic stroke (n)	Odds ratio (95% CI)	Intracerebral haemorrhage (n)	Ischaemic stroke (n)	Odds ratio (95% CI)	Intracerebral haemorrhage (n)	Ischaemic stroke (n)
≤0 mm Hg increase (reference group)	1·0 (0·5–2·1)	19	153	1·0 (0·5–2·2)	16	192	1·0 (0·6–1·5)	51	385
0–20 mm Hg increase	1·1 (0·5–2·4)	18	128	2·7 (1·3–5·7)	26	114	1·9 (1·0–3·3)	21	85
21–40 mm Hg increase	1·2 (0·6–2·5)	20	132	1·9 (0·9–4·1)	18	114	3·5 (1·7–7·3)	15	32
41–60 mm Hg increase	3·4 (1·7–6·8)	30	71	5·5 (2·5–12·0)	22	48	5·8 (2·4–13·4)	13	17
>60 mm Hg increase	5·4 (2·5–11·3)	26	39	11·1 (5·0–25·0)	26	28	24·5 (7·1–105·8)	13	4

Proportions of intracerebral haemorrhage versus ischaemic stroke stratified according to the increase between the first post-event SBP and premorbid levels. Numbers for 10-year average premorbid SBP do not add to total because data are for only patients with at least two measures of SBP during the past 10 years. SBP=systolic blood pressure.

Differences between ischaemic stroke and primary intracerebral haemorrhage in post-event systolic blood pressure were independent of a previous diagnosis of hypertension (table 2) and use of antihypertensive drugs (appendix p 8). Of ischaemic strokes categorised by the TOAST criteria, patients with small-vessel events had the highest mean post-event systolic blood pressure (table 2) and the greatest increase in the acute phase compared with mean premorbid systolic blood pressure (22·7 mm Hg). Similar differences were seen for deep or posterior intracerebral haemorrhage versus lobar bleeds (table 2).

All systolic blood pressure readings in the first 24 h in patients who presented within 90 min of onset are given in the appendix. Systolic blood pressure decreased more rapidly (p=0·0007) after primary intracerebral haemorrhage (decrease 1·7 mm Hg per h, 41·4 mm Hg in 24 h) than after ischaemic stroke (0·56 mm Hg per h, 13·6 mm Hg in 24 h). In these analyses, we excluded data for six patients with primary intracerebral haemorrhage who received additional blood pressure-lowering treatment within 24 h. The findings were unchanged in an additional analysis after exclusion of data for patients who died within the first 24 h.

We tested some of our findings on data from the Oxfordshire Stroke Community Project (OSCP), in which 65 patients had primary intracerebral haemorrhage and 294 had major ischaemic stroke (defined by modified Rankin Scale >2 at 1 month in the absence of baseline NIHSS scores). The time from stroke onset to first examination by the study neurologist was missing or was more than 7 days in 111 patients (22 with primary intracerebral haemorrhage, 89 with ischaemic stroke). Of the remaining 248 patients (appendix p 10), 219 (38 with primary intracerebral haemorrhage, 181 with ischaemic stroke) had their premorbid blood pressure recorded in primary care. Although the median time from stroke onset to blood pressure recording by the study neurologist was 2 days (IQR 0–4), differences between ischaemic stroke and primary intracerebral haemorrhage in OSCP were similar to those in our study. The mean first post-event systolic blood pressure was higher than the most recent pre-event level in patients with primary intracerebral haemorrhage (178·8 mm Hg [SD 33·6] *vs* 161·4 mm Hg [23·2]; p=0·0037) and was similar to the maximum premorbid systolic blood pressure (185·1 mm Hg [31·3]; p=0·30). However, the mean first post-event systolic blood pressure after ischaemic stroke was no higher than the most recent premorbid systolic blood pressure (156·5 mm Hg [29·2] *vs* 156·4 mm Hg [29·7]; p=0·97) and was lower than the mean maximum-recorded premorbid systolic blood pressure (184·4 mm Hg [35·1]; p<0·0001).

## Discussion

In patients with intracerebral haemorrhage in our study, blood pressure was substantially increased after stroke compared with usual premorbid levels. This increase was due both to a rise in premorbid systolic blood pressure in the days and weeks before the event and to a subsequent additional increase in systolic blood pressure from the last premorbid reading that was greater than that seen after ischaemic stroke. In patients with ischaemic stroke, acute post-event systolic blood pressure was much closer to the premorbid levels to which these patients were presumably accustomed, with no rise in systolic blood pressure before the event and only a small post-event increase. Consequently, in the first 24 h after stroke, blood pressure decreased much more after intracerebral haemorrhage than after ischaemic stroke.

To the best of our knowledge, this is the first prospective, population-based study of acute post-stroke hypertension and the first study to compare post-stroke readings with detailed premorbid blood pressure data (panel). Long-term hypertension is a major risk factor for primary intracerebral haemorrhage, accelerating small-vessel disease and increasing the risk of rupture.^41^ However, primary intracerebral haemorrhage can also occur during hypertensive crises and after major stress associated with short-term increase in blood pressure,^42^, ^43^ suggesting that, in at least some patients, acute post-stroke hypertension might partly reflect a recent, premorbid increase in blood pressure. Our findings substantiate this notion, but suggest that post-stroke factors also contribute to acute post-stroke hypertension, the first post-stroke blood pressure being higher than the most recent premorbid blood pressure even in patients with a recent pre-event rise. Intracerebral haemorrhage often causes acutely increased intracranial pressure,^41^ which would raise blood pressure via the Cushing reflex,^44^ and an increase in blood pressure of 20–60 mm Hg is seen during major psychological stress,^31^ which most probably also contributes to post-stroke levels. Indeed, it is surprising, therefore, that the increase in blood pressure over premorbid levels after ischaemic stroke is so limited, with most patients having had higher readings in the doctor's surgery in the past.

Panel **Research in context**

**Systematic review**

We searched Medline and Embase up to Oct 20, 2013, using the search terms “post-stroke hypertension” and “blood pressure in acute stroke”. We also reviewed the reference lists of retrieved studies for relevant papers. We restricted our search to studies done in human beings. We applied no language restrictions. Several studies have shown that blood pressure is often high immediately after stroke and that it usually decreases over the subsequent few days, irrespective of treatment. However, we identified no study that had compared post-stroke blood pressure with premorbid levels.

**Interpretation**

Our finding that blood pressure is raised compared with usual premorbid levels after intracerebral haemorrhage but not after major ischaemic stroke provides a potential explanation for why the risks and benefit of lowering blood pressure acutely after stroke might be expected to differ between intracerebral haemorrhage and major ischaemic stroke.

The distribution of blood pressures in the acute phase in patients with ischaemic stroke was broader than the distribution of the most recent readings (figure 2), and visit-to-visit variability in premorbid blood pressure was higher than in primary intracerebral haemorrhage, which is perhaps consistent with evidence that transient peaks and troughs in blood pressure can cause ischaemic stroke.^31^, ^42^, ^43^, ^45^, ^46^ Post-stroke blood pressure in patients with ischaemic stroke, however, seemed to mainly reflect longer-standing premorbid levels. The possible exceptions were patients with small-vessel ischaemic stroke, who had higher post-event levels than those with ischaemic stroke of other causes, which is consistent with previous findings,^6^, ^7^ and had a greater increase in systolic blood pressure compared with recent premorbid levels (small vessel stroke was the only subtype in which post-event blood pressure was similar to maximum premorbid levels). We saw similar associations for deep or posterior versus lobar primary intracerebral haemorrhage, which is also consistent with the hypothesis that hypertension is a particularly important risk factor for disease of the small perforating vessels.

Our findings have implications for interpretation of studies of acute blood pressure after stroke, particularly for apparent relations with outcome in observational studies without data on premorbid blood pressure levels.^47^, ^48^, ^49^ They also provide a potential physiological rationale to explain why the effects of acute blood pressure-lowering might differ in intracerebral haemorrhage 26, 27, 28 versus major ischaemic stroke.^20^, ^21^, ^22^, ^23^, ^24^, ^25^ The absence of benefit and possible harm associated with acute blood pressure-lowering in ischaemic stroke might be related to reduction of blood pressure to a level to which a patient is unaccustomed. In accustomed hypertension, the cerebral perfusion curve is shifted to the right,^50^ such that rapid blood pressure-lowering could compromise blood flow at a time when perfusion is already acutely compromised. Indeed, subgroup analyses in the SCAST trial^24^ suggested that blood pressure-lowering was harmful in patients with pre-existing hypertension, and blood pressure-lowering in INTERACT II^28^ also tended to be less effective in those with known prior hypertension. However, not all trials have shown this trend,^25^ and some uncertainty still remains as to the role of post-stroke blood pressure in early outcome. Our findings do, nevertheless, draw attention to the importance of consistent long-term control of blood pressure, especially in prevention of intracerebral haemorrhage, for which sustained increases in blood pressure for a short period seem to be a trigger.

Our study has some limitations. First, late presentation might have led to underestimation of the hyper-acute post-stroke blood pressure levels. However, we also did most analyses in patients presenting within 90 min and 3 h of the event and saw similar results to those in the overall analysis, and more finely stratified analyses (table 3) showed that differences between intracerebral haemorrhage and ischaemic stroke were greatest in the hyper-acute period. Second, post-stroke use of antihypertensive drugs might have affected our analysis of the post-stroke decrease in blood pressure levels. However, acute treatment was very rare and our analysis of the timecourse of post-stroke blood pressure excluded the few treated patients. Third, the accuracy of blood pressure readings can be affected by measurement error. However, observer error in nurses and physicians in blood pressure measurements has an SD of about 5 mm Hg and could not account for the difference we saw between intracerebral haemorrhage and ischaemic stroke. Fourth, a systematic protocol for recording of premorbid blood pressure was not possible, and we had to rely on measurements made in routine clinical practice in primary care. However, because our primary aim was to compare ischaemic stroke and intracerebral haemorrhage, for which the number and timing of premorbid measurements were very similar, this study limitation should not have introduced any bias. Moreover, most patients were older than 65 years, the age at which routine annual screening measurements of blood pressure are done in UK primary care, so that readings would be fairly standardised. Indeed, the extent of visit-to-visit variability in blood pressure is similar to that reported in previous randomised controlled trials.^39^, ^51^ Finally, we excluded patients with transient ischaemic attack or minor stroke, in whom acute-phase blood pressure might be higher than in those with major stroke. However, inclusion of these mainly ischaemic events would have introduced a severity bias in our comparison with intracerebral haemorrhage and would have made our study population very different to that included in previous randomised controlled trials of blood pressure-lowering in acute stroke.^20^, ^21^, ^22^, ^23^, ^24^, ^25^, ^26^, ^27^, ^28^

Our findings provide a potential rationale for why the risks and benefit of lowering blood pressure acutely after stroke might be expected to differ between intracerebral haemorrhage and major ischaemic stroke and have implications for design of future trials, which should take account of premorbid blood pressure in eligibility criteria and analyses. Our findings also draw attention to the need for consistent control of blood pressure in prevention of intracerebral haemorrhage.
