# Supplementary material for: Acute post-stroke blood pressure relative to premorbid levels in intracerebral haemorrhage versus major ischaemic stroke: a population-based study
Source: Lancet Neurol. 2014 Apr;13(4):374–84. doi: 10.1016/S1474-4422(14)70031-6 (PMC4238109; doi:10.1016/S1474-4422(14)70031-6)
Supplement: Supplementary appendix [file mmc1.pdf]

THE LANCET Neurology

Supplementary webappendix

This webappendix formed part of the original submission and has been peer reviewed. We post it as supplied by the authors.

Supplement to: Fischer U, Cooney MT, Bull LM, et al. Acute post-stroke blood pressure relative to premorbid levels in intracerebral haemorrhage versus major ischaemic stroke: a population-based study. *Lancet Neurol* 2014; published online Feb 28. http://dx.doi.org/10.1016/S1474-4422(14)70031-6.

Webappendix

P1. Supplemental methods of case ascertainment: hot and cold pursuit

Hot pursuit was based on the daily assessment of all patients with a possible vascular event identified by: 1) Daily searches of Emergency Department admission and symptom/diagnosis registers; 2) Daily listing from the central admissions department of all patients from our general practices admitted to hospital, and assessment of these patients in hospital; 3) Daily visits to all acute medical wards and stroke unit; 4) Provosion of daily study TIA and stroke clinic for participating primary care practices and review of all referrals to other similar clinics; 5) Daily identification via Bereavement Officers of patients dead on arrival at hospital or who died soon after; 6) Daily assessment of all patients undergoing diagnostic angiographic, angioplasty/stenting procedures.

The methods of cold pursuit were: 1) Frequent contact with general practices and monthly searches of computerised practice diagnostic codes; 2) Monthly practice-specific list of all patients with relevant diagnostic codes from the coding departments covering all acute and community hospitals (Hospital Episode Statistics – HES – data); 3) Monthly searchs of all requests for brain and carotid imaging; 4) Monthly visits to the Coroner’s Office to review out-of-hospital deaths; 5) Review of all death certificates and relevant clinical details in the study practices; 6) Practice-specific listings of all ICD-10 death codes from the local Department of Public Health;

P2. The distribution of stroke severity (NIHSS scores) at the time of first assessment in patients with intracerebral haemorrhage versus ischaemic stroke.

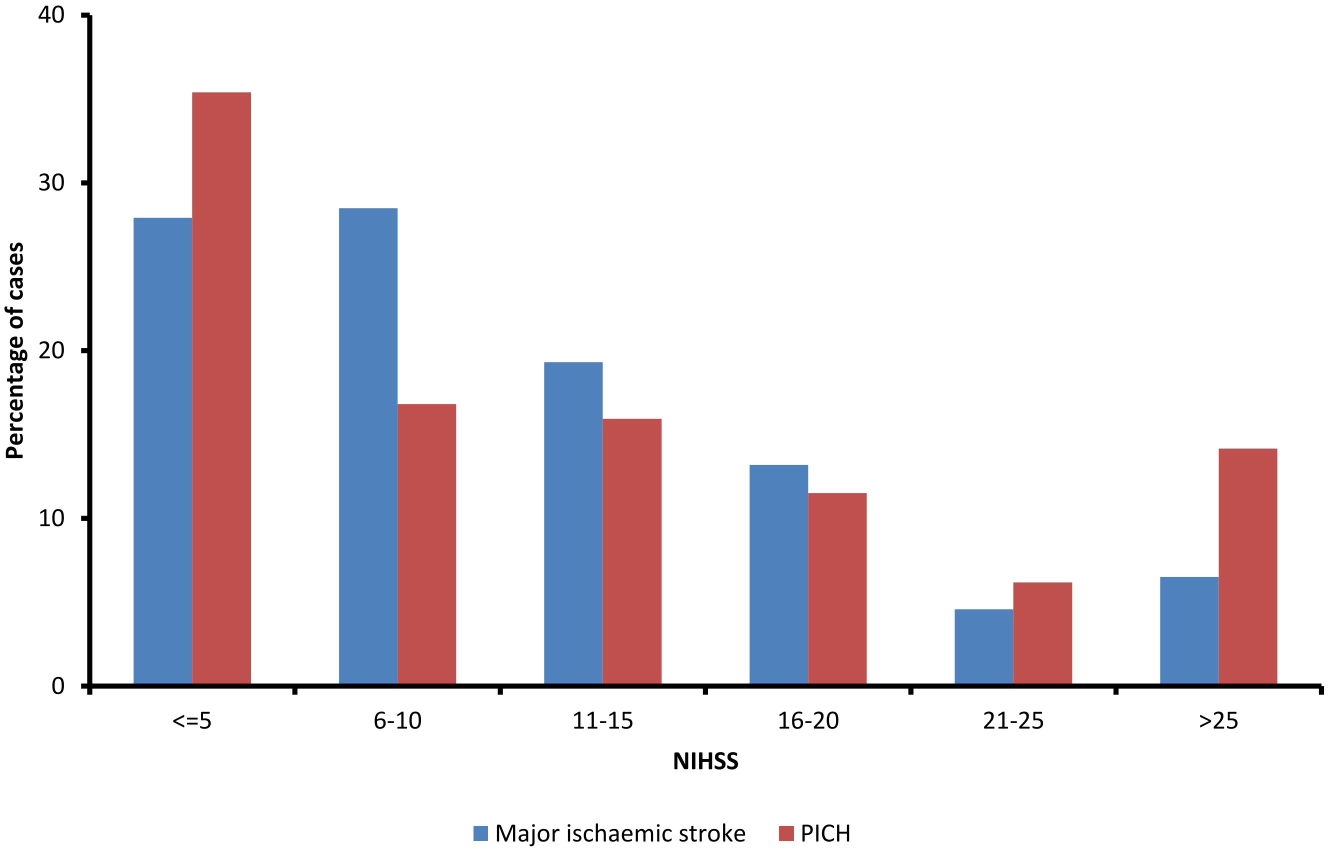

P3. The distribution of time from most recent pre-morbid BP reading and stroke onset in patients with P ICH and ischaemic stroke.

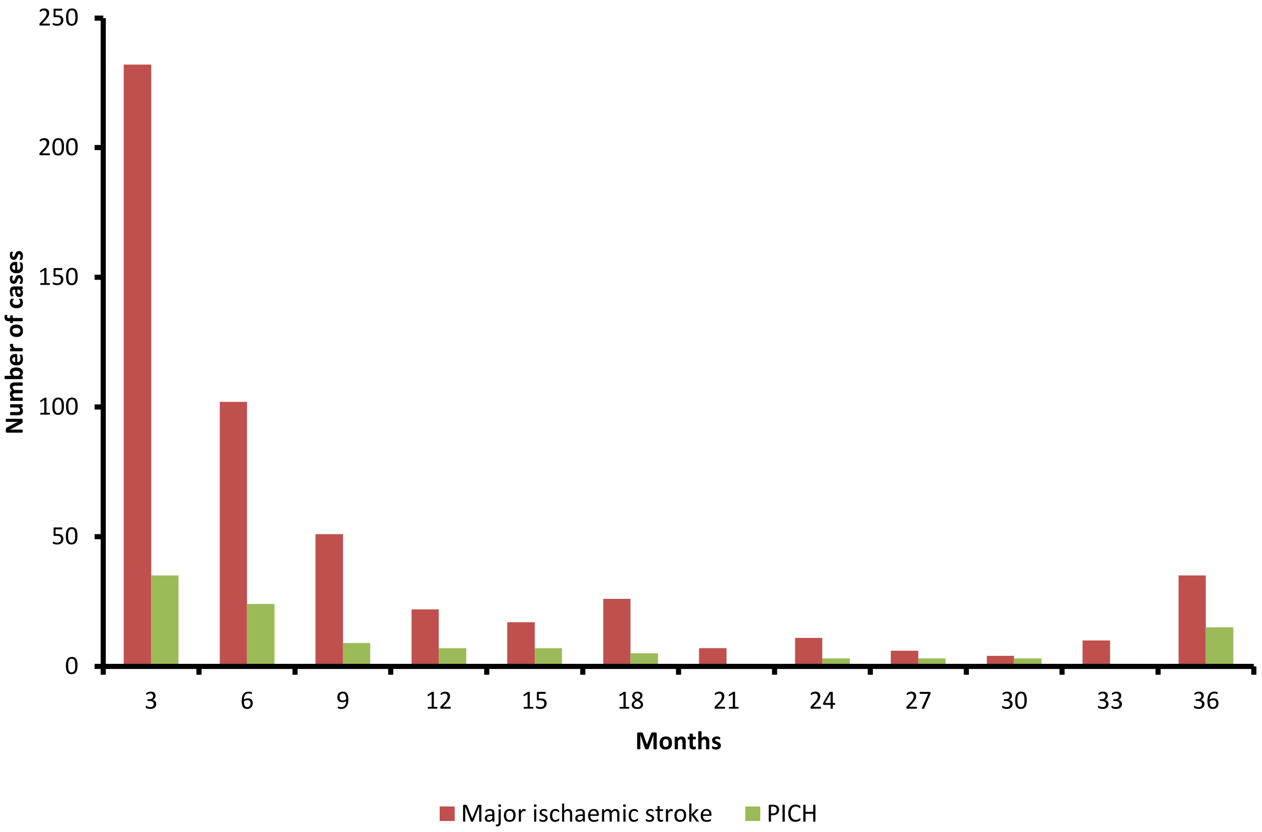

P4. The association between the number of premorbid BP readings and the mean pre-morbid SBP for patients with ICH versus ischaemic stroke. The trend lines and equations are derived from linear regression analyses.

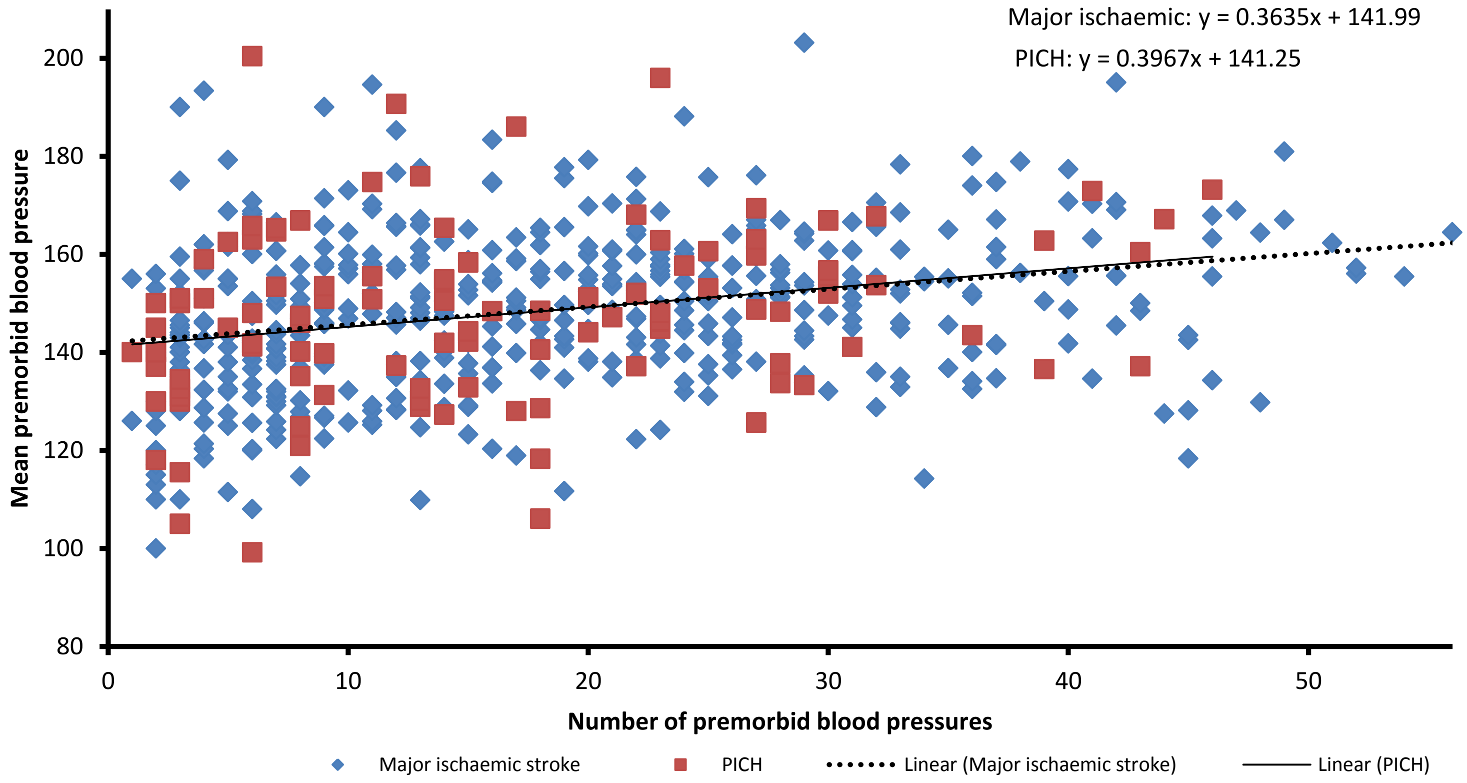

P5. Post event and pre-morbid SBPs in stroke patients assessed 3 hours or more after stroke onset.

	Patients assessed 3 hours or more after stroke				
	N	Maximum premorbid	10 year mean premorbid	Most recent premorbid	First post event
Major ischaemic stroke	229	182.5 (30.7)	141.0 (22.2)	149.4 (16.5)	157.6 (31.0)
Intracerebral haemorrhage	45	169.5 (28.3)	140.4 (21.1)	145.6 (17.4)	172.2 (34.0)
ICH LOCATION					
Lobar	19	165.8 (28.6)	145.4 (17.1)	140.3 (17.9)	169.2 (37.2)
Deep/Posterior	26	172.2 (28.3)	145.7 (17.9)	140.4 (23.5)	174.5 (32.1)
TOAST					
Cardioembolic	79	187.8 (30.3)	149.2 (16.3)	136.6 (21.7)	158.1 (32.5)
Large artery	11	186.7 (28.6)	155.2 (17.4)	157.4 (30.8)	175.9 (29.5)
Small vessel	34	177.8 (29.4)	149.3 (18.3)	147.2 (21.7)	167.8 (24.6)
Undetermined	37	183.7 (29.2)	150.7 (17.4)	142.6 (17.7)	163.3 (27.0)
Unknown	56	176.6 (33.6)	148.0 (16.1)	138.1 (22.9)	144.8 (32.9)
Multiple causes	7	186.6 (25.2)	148.4 (11.1)	146.3 (24.7)	144.9 (17.6)
Other known cause	5	175.8 (34.5)	148.5 (16.4)	143.0 (11.9)	158.2 (24.5)

P6. Sensitivity analyses differences between ICH and major ischaemic stroke in the first post-event SBP and in the first post-event \sbp minus the most recent pre-event SBP in: 1. All patients excluding those with wake-up strokes; 2. Patients with two pre-morbid SBP in last year and first post-event SBP within 6 hours of stroke onset.

1. Analyses in patients excluding wake-up strokes

		N	Mean	Std. Deviation	p-difference
First post Systolic	Major ischaemic stroke	377	157.87	30.678	<0.0001
	ICH	103	183.20	37.747	
Post - minus pre SBP	Major ischaemic stroke	377	16.1061	32.47101	<0.0001
	ICH	103	38.1553	37.81969	

2. Analyses confined to patients with two pre-morbid SBP in last year and first post-event SBP within 6 hours of stroke onset

		N	Mean	Std. Deviation	p-difference
Closest post Systolic	Major ischaemic stroke	273	160.31	30.513	0.001
	ICH	58	178.31	36.235	
Post minus pre SBP	Major ischaemic stroke	273	21.3187	30.11493	0.003
	ICH	58	35.8966	33.23953	

P7. The number and timing of all BP measurements during the first 24-hours post-stroke in the subset of patients with a first measurement within 90-minutes of stroke onset.

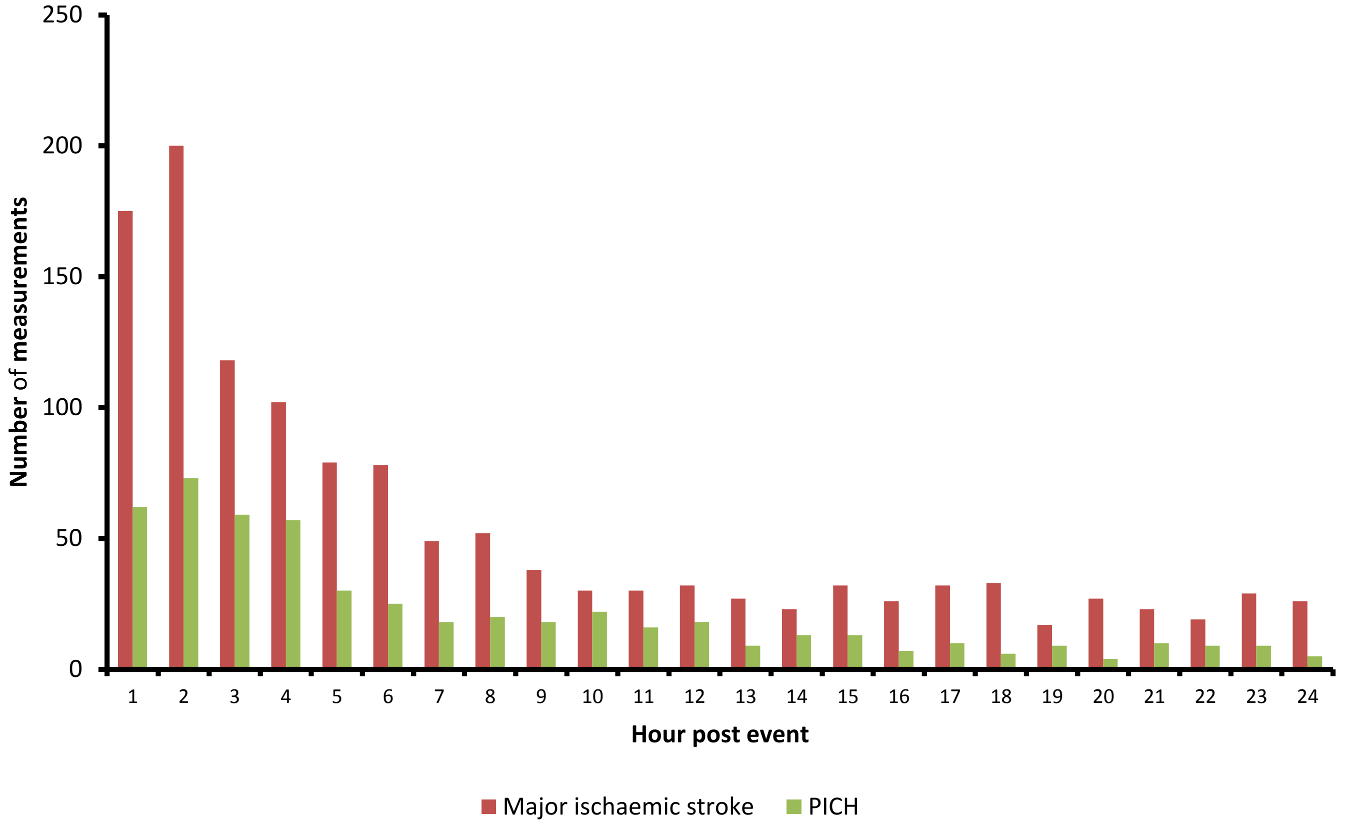

P8. Acute post event SBP and premorbid values in major ischaemic stroke and ICH patients, subdivided by pre-morbid use of antihypertensive drugs.

	Patients assessed <3 hours from stroke onset					All patients				
	N	Maximum premorbid	10 year mean premorbid	Most recent premorbid	First post event	N	Maximum premorbid	10 year mean premorbid	Most recent premorbid	First post event
**Not on antihypertensives**:<br>Major ischaemic stroke	92	163.4 (22.9)	143.9 (14.1)	138.1 (17.4)	159.2 (27.8)	171	164.2 (23.8)	143.8 (14.9)	137.9 (18.9)	158.8 (28.7)
Intracerebral haemorrhage	29	167.0 (25.7)	142.6 (17.0)	144.2 (21.2)	193.4 (37.4)	59	163.0 (25.7)	141.9 (16.9)	142.5 (19.1)	181.7 (36.9)
**On antihypertensives**:<br>Major ischaemic stroke	202	187.1 (27.9)	149.5 (15.4)	141.7 (21.8)	158.2 (31.2)	352	189.0 (28.7)	150.7 (15.8)	142.1 (22.2)	157.7 (31.4)
Intracerebral haemorrhage	39	185.7 (23.7)	153.5 (17.4)	147.9 (23.6)	187.1 (39.5)	54	187.0 (23.0)	153.6 (16.7)	145.6 (24.9)	184.1 (38.7)

P9. Repeated SBP measurements in patients assessed within 90 minutes of stroke onset. Time is hours from stroke onset. The red line and associated equations are derived from linear regression.

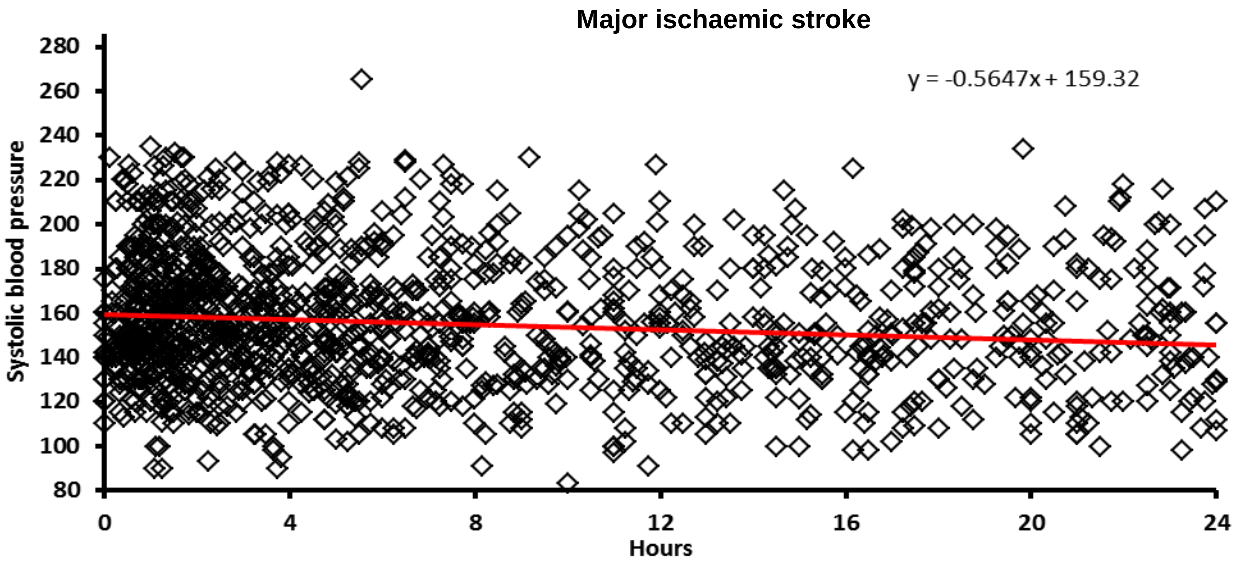

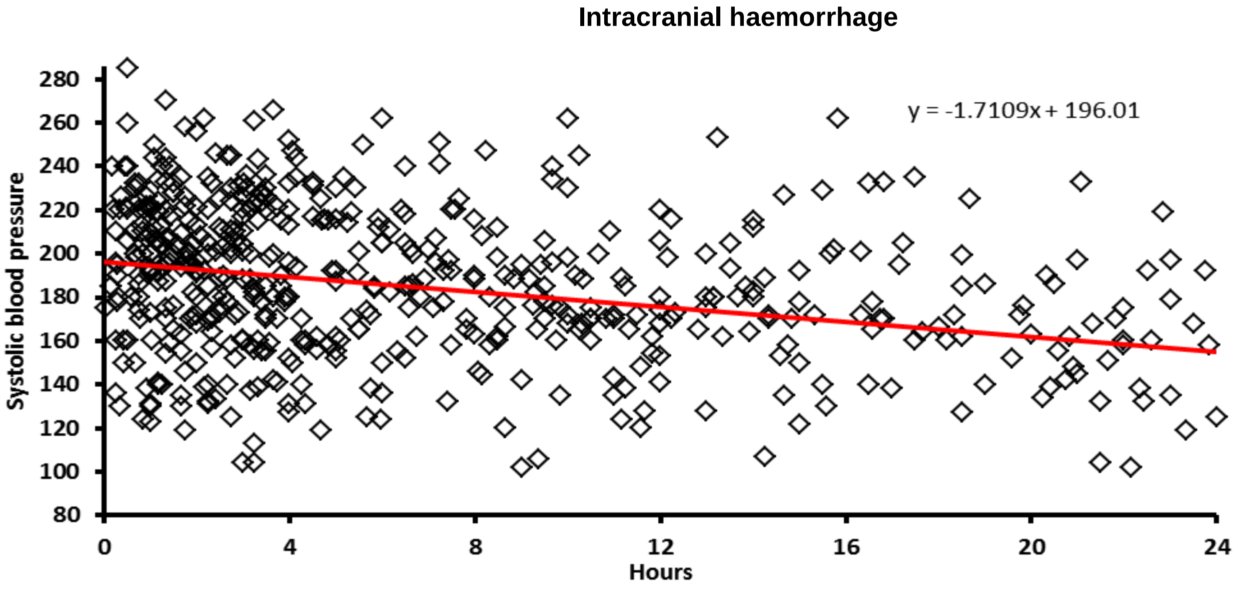

P10. Baseline characteristics of patients with major ischaemic stroke and ICH: OCSP population

Characteristics	Major ischaemic stroke	Intracerebral haemorrhage
	n = 181	n = 38
Age (mean)	77.0	72.4
Female sex	106 (59%)	22 (58%)
Vascular risk factors		
Hypertension	91 (51%)	22 (58%)
Diabetes mellitus	22 (12%)	0
Smoking		
Never smoked	65 (38%)	14 (40%)
Ex-Smoker	45 (26%)	7 (20%)
Current Smoker	63 (36%)	14 (40%)
Previous atrial fibrillation	31 (17%)	5 (14%)
Previous myocardial infarction	36 (20%)	4 (11%)
Previous angina	30 (17%)	2 (5%)
Previous transient ischaemic attack	28 (15%)	3 (8%)
Previous peripheral vascular disease	23 (13%)	1 (3%)
Medication		
Prior antihypertensives	41 (30%)	3 (8%)
Prior antiplatelet	6 (3%)	3 (8%)
Type of event		
Incident event	100%	100%
Recurrent event	0%	0%
